# Influences, Barriers, and Facilitators to COVID-19 Vaccination: Cross-sectional Survey on Vaccine Hesitancy in 2 Rural States

**DOI:** 10.2196/39109

**Published:** 2022-12-01

**Authors:** Elaine Nguyen, Melanie Wright, John Holmes, Kevin Cleveland, Catherine Oliphant, Mary Nies, Renee Robinson

**Affiliations:** 1 Department of Pharmacy Practice and Administration College of Pharmacy Idaho State University Meridian, ID United States; 2 Department of Pharmacy Practice and Administration College of Pharmacy Idaho State University Pocatello, ID United States; 3 School of Nursing College of Health Pocatello, ID United States; 4 Department of Pharmacy Practice and Administration College of Pharmacy University of Alaska/Idaho State University Anchorage, AK United States

**Keywords:** COVID-19, COVID-19 vaccines, vaccine hesitancy, cross-sectional studies, rural populations

## Abstract

**Background:**

Vaccination remains one of the most effective ways to limit the spread of infectious diseases such as that caused by severe acute respiratory syndrome coronavirus 2 (SARS-CoV-2), the virus responsible for COVID-19. Unfortunately, vaccination hesitancy continues to be a threat to national and global health. Further research is necessary to determine the modifiable and nonmodifiable factors contributing to COVID-19 vaccine hesitancy in under-resourced, underserved, and at-risk rural and urban communities.

**Objective:**

This study aimed to identify, understand, and address modifiable barriers and factors contributing to COVID-19 vaccine hesitancy among vaccine-eligible individuals with access to the vaccine in Alaska and Idaho.

**Methods:**

An electronic survey based on the World Health Organization (WHO) Strategic Advisory Group on Experts (SAGE) on Immunization survey tool and investigators’ previous work was created and distributed in June 2021 and July 2021. To be eligible to participate in the survey, individuals had to be ≥18 years of age and reside in Alaska or Idaho. Responses were grouped into 4 mutually exclusive cohorts for data analysis and reporting based on intentions to be vaccinated. Respondent characteristics and vaccine influences between cohorts were compared using Chi-square tests and ANOVA. Descriptive statistics were also used.

**Results:**

There were data from 736 usable surveys with 40 respondents who did not intend to be vaccinated, 27 unsure of their intentions, 8 who intended to be fully vaccinated with no doses received, and 661 fully vaccinated or who intended to be vaccinated with 1 dose received. There were significant differences in characteristics and influences between those who were COVID-19 vaccine-hesitant and those who had been vaccinated. Concerns related to possible side effects, enough information on long-term side effects, and enough information that is specific to the respondent’s health conditions were seen in those who did not intend to be fully vaccinated and unsure about vaccination. In all cohorts except those who did not intend to be fully vaccinated, more information about how well the vaccine works was a likely facilitator to vaccination.

**Conclusions:**

These survey results from 2 rural states indicate that recognition of individual characteristics may influence vaccine choices. However, these individual characteristics represent only a starting point to delivering tailored messages that should come from trusted sources to address vaccination barriers.

## Introduction

Immunization is the greatest public health achievement of all time, saving over 3 million lives worldwide each year [1,2]. State and national immunization programs have been so successful that many Americans view the risks of vaccine-preventable diseases such as measles, pertussis, and polio as minimal [1]. Vaccination remains one of the most effective ways to limit the spread of infectious diseases such as that caused by severe acute respiratory syndrome coronavirus 2 (SARS-CoV-2), the virus responsible for COVID-19 [3]. However, waning public confidence in vaccines, especially the COVID-19 vaccine, remains a cause for concern [4-9]. In addition, believed threshold requirements for vaccination to achieve herd immunity have shifted and increased with new variants [10,11]. As of August 2022, ~67% of the total population had been fully vaccinated (ie, primary series completed) [12], but there continues to be a need for both primary series completion and booster doses [13].

Vaccine hesitancy, one of the top 10 threats to global health, is the delay or refusal to receive a vaccine, despite access, availability, and perceived effectiveness of the vaccine [5]. Preliminary research suggests that health decisions, such as to receive or not receive a vaccine, are highly influenced by social and cultural factors (eg, political ideology, past experiences with health services, family histories, the moral dilemma between individual autonomy and the greater public health) [6,13,14]. In addition, several other complex factors may be contributing to the increased hesitancy that has been noted with the COVID-19 vaccine [5,8,15].

Given the importance of this topic, researchers have been seeking to better understand acceptability of COVID-19 vaccination and drivers of hesitancy. Global surveys conducted in different countries have shown concerns for vaccine safety and effectiveness [16-21]. At the beginning of the pandemic in May 2020, an online survey of Americans found that 69% of respondents were willing to receive a COVID-19 vaccine [16]. There were statistically significant differences in those willing and not willing to get vaccinated based on how well the vaccine works and the number of people infected with COVID-19. Since this initial survey was conducted, the COVID-19 vaccination landscape has continued to shift in the United States and globally.

With complex factors impacting vaccination decisions, further research is necessary to determine the modifiable and nonmodifiable factors contributing to COVID-19 vaccine hesitancy in under-resourced, underserved, and at-risk rural and urban communities. The goal of this project was to identify, understand, and address modifiable barriers and factors contributing to COVID-19 vaccine hesitancy among vaccine-eligible individuals with access to the vaccine in Alaska and Idaho. The primary goal of this paper was to present the results from a vaccine hesitancy survey.

## Methods

### Survey Details

The survey was based on the World Health Organization (WHO) Strategic Advisory Group on Experts (SAGE) on Immunization survey tool and investigators’ previous qualitative work with residents of Alaska and Idaho who remain hesitant to receive the COVID-19 vaccine [1,22,23]. Some survey questions from the WHO SAGE were previously validated, and some were from field experts. The draft survey underwent several revisions and was reviewed multiple times by project investigators (n=7) and the project advisory board. The advisory board (n=11) was composed of community members, health care providers, and public health organization representatives. Although the final survey was extensively reviewed, it did not undergo any formal validation studies.

The survey (Multimedia Appendix 1) was estimated to take 10 minutes and included 31 questions divided into 4 parts: introduction, barriers and facilitators, influences, and demographics. All questions, except those in the introduction (eligibility screening and vaccination status), were optional. To be eligible to participate in the survey, individuals had to be ≥18 years of age and reside in Alaska or Idaho. The focus on this population was to capture adult perspectives from primarily rural states.

The survey was created and made available via Qualtrics online survey software. It was distributed through project investigators and advisory board member contacts. It was also promoted in Facebook advertisements. A broad defined audience was used for the Facebook advertisements, with advertisements targeting only location (Alaska or Idaho) and age (≥18 years). The survey was available for approximately 1 month, with responses collected from June 11, 2021, through July 16, 2021.

### Data Analysis

Given the broad survey distribution, responses were reviewed for validity, and responses deemed potentially invalid were removed from analysis. Responses were removed if at least one of the following criteria were met: The survey was not finished, completed multiple times from the same IP address with no unique free-text responses, or not completed in Alaska and Idaho (as determined by GPS coordinates). IP address and GPS data are automatically collected in the Qualtrics survey platform.

Eligible and valid responses were included in the data analysis. Respondents were grouped into 4 mutually exclusive cohorts: (1) did not intend to be fully vaccinated, (2) were unsure of their vaccination intentions, (3) intended to be fully vaccinated but had not yet received their first dose, and (4) were fully vaccinated or intended to be fully vaccinated and had received at least 1 dose. When comparing respondent characteristics and vaccine influences between cohorts, Chi-square tests (for nominal data) and ANOVA (for continuous data) were used. *P* values <.05 were considered statistically significant when comparing respondent characteristics. When comparing vaccine influences, a Bonferroni correction with a *P* value <.007 was considered statistically significant. Descriptive statistics (counts and percentages) were used to describe barriers, facilitators, and trust in sources for vaccine information. Cronbach alpha was also used to measure reliability for barriers, facilitators, and trust in sources for vaccine information.

### Ethical Considerations

This work underwent an expedited review and was approved by the Idaho State University Institutional Review Board (IRB-FY2021-256). Respondents indicated their consent to participate after reading survey background information (eg, purpose, estimated completion time) and by continuing to the next survey page.

After determining if a response was valid and categorizing the response into a mutually exclusive cohort, any individual response data not associated with survey responses were removed for analysis to protect respondent privacy. To incentivize participation, a raffle to be entered to win one of 20 US $100 Amazon electronic gift cards was offered. To maintain respondent privacy, raffle information was collected in a separate form to keep survey responses anonymous.

## Results

After removal of invalid responses, there were data from 736 usable surveys: 40 respondents did not intend to be vaccinated, and 27 were unsure of their intentions. Although 8 respondents had not yet received any COVID-19 vaccine, they had intended to be fully vaccinated. Lastly, 661 respondents were fully vaccinated (n=654) or intended to be fully vaccinated with 1 dose received (n=7). Characteristics of survey respondents are presented in Table 1. There were statistically significant differences across cohorts among all characteristics evaluated (see *P* values in Table 1).

The intended to be vaccinated cohort with no doses received had the lowest mean age (33.3 years), but this sample size was small. Those who were fully vaccinated or intended to be fully vaccinated with at least 1 dose received had the oldest mean age (59.1 years). Of those who did not intend to be vaccinated, the lowest age of a respondent was 35 years, whereas the other cohorts had younger respondents. The distribution of respondents’ ages by cohorts is available in Multimedia Appendix 2.

Nearly 90% (7/8, 88%) of respondents in the intended to be fully vaccinated but had not yet received their first dose cohort were men versus only 18.0% (119/661) in the fully vaccinated/intended to be fully vaccinated with 1 dose received cohort. One-half (20/40, 50%) of those who did not intend to be fully vaccinated identified as Christian, whereas only 34.5% (231/669) of those who intended to be fully vaccinated or were already fully vaccinated were Christian. Conversely, a reverse pattern was seen across these cohorts with those who were agnostic, atheist, or believed in nothing in particular. In those who did not intend to be vaccinated, the largest political preference was Republican (17/40, 43%). In those fully vaccinated/intended to be fully vaccinated with 1 dose received, the largest political preference was Democrat (314/661, 47.5%). Across cohorts, there were statistical differences in race and ethnicity, but overall, the groups were predominantly White and not Hispanic nor Latino. The majority of respondents had health insurance (702/736, 95.4%), with only 22 (22/736, 3.0%) reporting no insurance; 12 (12/736, 1.6%) were unsure or chose not to share their health insurance status.

When assessing vaccine influences, there continued to be differences across cohorts (Table 2). Only 25% (10/40) of those who did not intend to be vaccinated had a medium-high perceived risk of getting COVID-19 versus 43.0% (284/661) of those fully vaccinated/intended to be fully vaccinated. Interestingly, the percentage of those who had prior COVID-19 and had been really sick was twice as high in those with no plans of vaccination versus those fully vaccinated/intended to be fully vaccinated (5/40, 13% vs 39/661, 5.9%). Of the respondents, 90.2% (664/736) knew somebody who had COVID-19, and nearly one-third (239/736, 32.5%) knew somebody who had died from the disease. The proportion of those knowing somebody who died from COVID-19 was lower in those who did not intend to be vaccinated than in the other cohorts. Significant differences were seen in beliefs that vaccines work to prevent diseases and, related to this belief, the typical receipt of the influenza vaccine.

Data represented in Tables 3-5 represent descriptive statistics only. From these data, concerns related to possible side effects, enough information on long-term side effects, and enough information that is specific to respondents’ health conditions were seen in those who did not intend to be fully vaccinated and unsure about vaccination (Table 3). Practical factors for vaccination (ie, scheduling, time away from daily responsibilities for vaccination and side effects, child supervision) were not seen as barriers. The Cronbach alpha for barriers was 0.8184. In all cohorts except those who did not intend to be fully vaccinated, more information about how well the vaccine works is a likely facilitator to vaccination (Table 4). Factors such as payment to get the vaccine or requirements for work or travel were not likely facilitators or motivators to vaccination across all cohorts. The Cronbach alpha for facilitators was 0.7279.

In those not planning to receive the vaccine, there was low trust from most sources of information (Table 5). In those unsure, a primary care provider or doctor and pharmacist were the most trusted sources of information. This trend was also seen in those who intended to be fully vaccinated or those already fully vaccinated. The Cronbach alpha for trust in sources of vaccine information was 0.8239.

**Table 1:** Respondent characteristics.

Characteristic			Did NOT intend to be fully vaccinated (n=40)		Unsure of full vaccination intentions (n=27)		Intended to be fully vaccinated with no doses received (n=8)		Fully vaccinated OR intended to be with 1 dose received (n=661)		All (N=736)		*P* value
Age (years), mean (SD)			56.6 (11.0)		54.9 (15.6)		33.3 (12.0)		59.1 (14.5)		58.4 (14.7)		<.001
Age (years), median (range)			55.0 (35-92)		55.0 (25-80)		29.5 (23-61)		63.0 (18-85)		62.0 (18-92)		—^a^
**Gender identity, n (%)**													
	Man	12 (30.0)		4 (14.8)		7 (87.5)		119 (18.0)		142 (19.3)		.001	
	Woman	26 (65.0)		22 (81.5)		1 (12.5)		531 (80.3)		580 (78.8)			
	Other	1 (2.5)		0 (0)		0 (0)		5 (0.8)		6 (0.8)			
	Prefer not to say/no response	1 (2.5)		1 (3.7)		0 (0)		6 (1.0)		8 (1.1)			
**Race^b^, n (%)**													
	Alaska Native	0 (0)		1 (3.4)		1 (12.5)		1 (0.2)		3 (0.4)		<.001	
	American Indian/Native American	2 (5.0)		1 (3.4)		0 (0)		7 (1.1)		10 (1.4)			
	Asian	0 (0)		0 (0)		0 (0)		9 (1.4)		9 (1.2)			
	Black/African American	0 (0)		0 (0)		2 (25.0)		2 (0.3)		4 (0.5)			
	Native Hawaiian/Pacific Islander	0 (0)		0 (0)		0 (0)		2 (0.3)		2 (0.3)			
	White	34 (85.0)		26 (89.7)		5 (62.5)		633 (95.8)		698 (94.8)			
	Other	2 (5.0)		0 (0)		0 (0)		7 (1.1)		9 (1.2)			
	Prefer not to say/no response	2 (5.0)		1 (3.4)		1 (12.5)		13 (2.0)		17 (2.3)			
**Ethnicity, n (%)**													
	Hispanic/Latino	1 (2.5)		3 (11.1)		1 (12.5)		12 (1.8)		17 (2.3)		.009	
	Not Hispanic/Latino	29 (72.5)		19 (70.4)		6 (75.0)		547 (82.8)		601 (81.7)			
	Other	1 (2.5)		1 (3.7)		0 (0)		43 (6.5)		45 (5.1)			
	Prefer not to say/no response	9 (22.5)		4 (14.8)		1 (12.5)		59 (8.9)		73 (9.9)			
**State, n (%)**													
	Alaska	0 (0)		5 (18.5)		5 (62.5)		15 (2.3)		25 (3.4)		<.001	
	Idaho	40 (100)		22 (81.5)		3 (37.5)		646 (97.7)		711 (96.6)			
**Religion^b^, n (%)**													
	Agnostic/atheist/nothing in particular	3 (7.5)		5 (18.5)		2 (25.0)		227 (34.3)		237 (32.2)		<.001	
	Christian	20 (50.0)		11 (40.7)		5 (62.5)		226 (34.2)		262 (35.6)			
	Jewish	1 (2.5)		0 (0)		0 (0)		6 (0.9)		7 (1.0)			
	Mormon	5 (12.5)		6 (22.2)		0 (0)		109 (16.5)		120 (16.3)			
	Muslim	1 (2.5)		0 (0)		1 (12.5)		0 (0)		2 (0.3)			
	Roman Catholic	3 (7.5)		3 (11.1)		0 (0)		56 (8.5)		62 (8.4)			
	Other	2 (5.0)		2 (7.4)		0 (0)		37 (5.6)		41 (5.6)			
	Prefer not to say/no response	6 (15.0)		3 (11.1)		0 (0)		38 (5.7)		47 (6.4)			
**Political preference^b^, n (%)**													
	Democrat	2 (5.0)		5 (18.5)		1 (12.5)		314 (47.5)		322 (42.8)		<.001	
	Republican	17 (42.5)		7 (25.9)		4 (50.0)		110 (16.6)		138 (18.8)			
	Independent	9 (22.5)		7 (25.9)		3 (37.5)		193 (29.2)		212 (28.8)			
	Other	2 (5.0)		3 (11.1)		0 (0)		51 (7.7)		56 (7.6)			
	Prefer not to say/no response	10 (25.0)		7 (25.9)		0 (0)		55 (8.3)		72 (9.8)			
**Highest grade finished/degree received, n (%)**													
	High school graduate/GED or less	2 (5.0)		1 (3.7)		1 (12.5)		26 (3.9)		30 (4.1)		.003	
	Some college, no degree/associate degree	16 (40.0)		12 (44.4)		2 (25.0)		150 (22.7)		180 (24.5)			
	Bachelor degree	11 (27.5)		4 (14.8)		2 (25.0)		226 (34.2)		243 (33.0)			
	Postbachelor degree	9 (22.5)		6 (22.2)		3 (37.5)		242 (36.6)		260 (35.3)			
	Other	2 (5.0)		4 (14.8)		0 (0)		14 (2.1)		20 (2.7)			
	Prefer not to say/no response	0 (0)		0 (0)		0 (0)		3 (0.5)		3 (0.4)			
**Employment status^b^, n (%)**													
	Employed by government (local, state, and federal)	4 (10.0)		3 (11.1)		3 (37.5)		106 (16.0)		116 (15.8)		<.001	
	Employed by a private company (for-profit and nonprofit)	12 (30.0)		6 (25.9)		4 (50.0)		130 (19.7)		152 (20.7)			
	Other	18 (45.0)		17 (63.0)		1 (12.5)		441 (66.7)		477 (64.8)			
	Prefer not to say/no response	8 (20.0)		2 (7.4)		0 (0)		11 (1.7)		21 (2.9)			

^a^Not calculated.

^b^Respondents could select all that apply; the sum of the percentages may be >100.

**Table 2:** Factors influencing the decision to be vaccinated.

Influence		Did NOT intend to be fully vaccinated (n=40), n (%)	Unsure of full vaccination intentions (n=27), n (%)	Intended to be fully vaccinated with no doses received (n=8), n (%)	Fully vaccinated OR intended to be with 1 dose received (n=661), n (%)	All (N=736), n (%)	*P* value
**Perceived risk of getting COVID-19**							
	None	9 (22.5)	0 (0)	1 (12.5)	37 (5.6)	47 (6.4)	.004
	Low	21 (52.5)	15 (55.6)	5 (62.5)	340 (51.4)	381 (51.8)	
	Medium	9 (22.5)	9 (33.3)	1 (12.5)	198 (30.0)	217 (29.5)	
	High	1 (2.5)	3 (11.1)	1 (12.5)	86 (13.0)	91 (12.4)	
**Perceived risk of getting really sick from COVID-19**							
	None	11 (27.5)	4 (14.8)	0 (0)	53 (8.0)	68 (9.2)	—^a^
	Low	20 (50.0)	10 (37.0)	4 (50.0)	283 (42.8)	317 (43.1)	
	Medium	7 (17.5)	6 (22.2)	1 (12.5)	199 (30.1)	213 (28.9)	
	High	2 (5.0)	7 (25.9)	3 (37.5)	125 (18.9)	137 (18.6)	
	No response	0 (0)	0 (0)	0 (0)	1 (0.2)	1 (0.1)	
**Prior COVID-19**							
	No	20 (50.0)	10 (37.0)	6 (75.0)	498 (75.3)	534 (72.6)	<.001
	Unsure	10 (25.0)	9 (33.3)	0 (0)	76 (11.5)	95 (12.9)	
	Yes, minor/no symptoms	4 (10.0)	5 (18.5)	2 (25.0)	46 (7.0)	57 (7.7)	
	Yes, really sick	5 (12.5)	3 (11.1)	0 (0)	39 (5.9)	47 (6.4)	
	No response	1 (2.5)	0 (0)	0 (0)	2 (0.3)	3 (0.4)	
**Known somebody who had COVID-19, worst outcome**							
	No	5 (12.5)	1 (3.7)	3 (37.5)	41 (6.2)	50 (6.8)	<.001
	Unsure	0 (0)	2 (7.4)	0 (0)	18 (2.7)	20 (2.7)	
	Yes, only minor/no symptoms	18 (45.0)	7 (25.9)	3 (37.5)	121 (18.3)	149 (20.2)	
	Yes, really sick	12 (30.0)	7 (25.9)	0 (0.0)	257 (38.9)	276 (37.5)	
	Yes, died	4 (10.0)	10 (37.0)	2 (25.0)	223 (33.7)	239 (32.5)	
	No response	1 (2.5)	0	0	1 (0.2)	2 (0.3)	
**Belief that vaccines work to prevent diseases**							
	Not at all	4 (10.0)	0 (0)	0 (0)	0 (0)	4 (0.5)	<.001
	A little	8 (20.0)	6 (22.2)	0 (0)	5 (0.8)	19 (2.6)	
	A moderate amount	14 (35.0)	5 (18.5)	4 (50.0)	47 (7.1)	70 (9.5)	
	A lot	13 (32.5)	16 (59.3)	4 (50.0)	608 (92.0)	641 (87.1)	
	No response	1 (2.5)	0 (0)	0 (0)	1 (0.2)	2 (0.3)	
**Typical receipt of flu vaccine**							
	No	31 (77.5)	13 (48.1)	1 (12.5)	92 (13.9)	137 (18.6)	<.001
	Unsure	0 (0)	1 (3.7)	2 (25.0)	7 (1.1)	10 (1.4)	
	Yes	8 (20.0)	12 (44.4)	5 (62.5)	557 (84.3)	582 (79.1)	
	Prefer not to say/no response	1 (2.5)	1 (3.7)	0 (0)	5 (0.8)	7 (1.0)	
**Worrisome allergies with COVID-19 vaccine**							
	No	26 (65.0)	12 (44.4)	3 (37.5)	588 (89.0)	629 (85.5)	<.001
	Unsure	6 (15.0)	3 (11.1)	2 (25.0)	27 (4.1)	38 (5.2)	
	Yes	5 (12.5)	12 (44.4)	3 (37.5)	42 (6.4)	62 (8.4)	
	Prefer not to say	3 (7.5)	0 (0)	0 (0)	4 (0.6)	7 (1.0)	

^a^Not performed due to low cell counts.

**Table 3:** Barriers to vaccination.

Barrier^a^	Did NOT intend to be fully vaccinated (n=40), n (%)			Unsure of full vaccination intentions (n=27), n (%)			Intended to be fully vaccinated with no doses received (n=8), n (%)			Fully vaccinated OR intended to be with 1 dose received (n=661), n (%)	
	Not at all/ little	Moderately/a lot	Not at all/ little		Moderately/a lot	Not at all/ little		Moderately/a lot	Not at all/ little		Moderately/a lot
Enough trusted information about the vaccine	18 (45.0)	22 (55.0)	8 (29.6)		18 (66.7)	1 (12.5)		7 (87.5)	573 (86.7)		87 (13.2)
Enough information about the vaccine in respondent language	30 (75.0)	10 (25.0)	19 (70.4)		7 (25.9)	3 (37.5)		5 (62.5)	611 (92.4)		43 (6.5)
Enough information on short-term vaccine side effects	22 (55.0)	18 (45.0)	8 (29.6)		18 (66.7)	3 (37.5)		5 (62.5)	560 (84.7)		95 (14.4)
Enough information on long-term vaccine side effects	11 (27.5)	29 (72.5)	4 (14.8)		22 (81.5)	2 (25.0)		6 (75.0)	519 (78.5)		134 (20.3)
Enough information about the vaccine that is specific to respondent’s health conditions	13 (32.5)	27 (67.5)	6 (22.2)		21 (77.8)	2 (25.0)		6 (75.0)	552 (83.5)		96 (14.5)
Process of scheduling a vaccine appointment	40 (100)	0 (0)	25 (92.6)		1 (3.7)	5 (62.5)		3 (37.5)	518 (78.4)		142 (21.5)
Possible side effects from the vaccine	8 (20.0)	32 (80.0)	5 (18.5)		22 (81.5)	0 (0)		8 (100)	596 (90.2)		61 (9.2)
Time it takes to get the vaccine	39 (97.5)	1 (2.5)	23 (85.2)		3 (11.1)	3 (37.5)		5 (62.5)	603 (91.2)		52 (7.9)
Time off needed from daily responsibilities if side effects were experienced	26 (65.0)	14 (35.0)	17 (63.0)		9 (33.3)	3 (37.5)		5 (62.5)	587 (88.8)		68 (10.3)
Child supervision^b^	30 (75.0)	1 (2.5)	21 (77.8)		2 (7.4)	7 (87.5)		1 (12.5)	434 (65.7)		14 (2.1)

^a^There are no responses that are not shown in the table.

^b^Not applicable for all respondents

**Table 4:** Facilitators to vaccination.

Facilitator^a^	Did NOT intend to be fully vaccinated (n=40), n (%)			Unsure of full vaccination intentions (n=27), n (%)			Intended to be fully vaccinated with no doses received (n=8), n (%)			Fully vaccinated OR intended to be with 1 dose received (n=661), n (%)	
	Not at all/ little	Moderately/a lot	Not at all/ little		Moderately/a lot	Not at all/ little		Moderately/a lot	Not at all/ little		Moderately/a lot
Somebody trusted tells to get the vaccine	39 (97.5)	1 (2.5)	21 (77.8)		6 (22.2)	1 (12.5)		7 (87.5)	427 (64.6)		227 (34.3)
Interaction with other people who are at high risk of getting really sick from COVID-19	36 (90.0)	4 (10.0)	15 (55.6)		12 (44.4)	2 (25.0)		6 (75.0)	294 (44.5)		361 (54.6)
People around the respondent get the vaccine	40 (100)	0 (0)	23 (85.2)		4 (14.8)	3 (37.5)		5 (62.5)	428 (64.8)		224 (33.9)
More information about how well the vaccine works	35 (87.5)	5 (12.5)	13 (48.1)		14 (51.9)	0 (0)		8 (100)	207 (31.3)		450 (68.1)
Getting the vaccine at primary care provider’s office	37 (92.5)	1 (2.5)	18 (66.7)		9 (33.3)	3 (37.5)		5 (62.5)	568 (85.9)		85 (12.9)
Getting the vaccine close to respondent	37 (92.5)	1 (2.5)	19 (70.4)		8 (29.6)	3 (37.5)		5 (62.5)	258 (39.0)		395 (59.8)
Paid to get the vaccine	38 (95.0)	1 (2.5)	24 (88.9)		3 (11.1)	3 (37.5)		5 (62.5)	645 (97.6)		11 (1.7)
Required for work	37 (92.5)	2 (5.0)	18 (66.7)		9 (33.3)	0 (0)		8 (100)	634 (95.9)		20 (3.0)
Required for travel	39 (97.5)	0 (0)	19 (70.4)		7 (25.9)	1 (12.5)		7 (87.5)	559 (84.6)		93 (14.1)
No longer have to wear a mask	38 (95.0)	1 (2.5)	19 (70.4)		7 (25.9)	0 (0)		8 (100)	462 (69.9)		193 (29.2)

^a^There are no responses that are not shown in the table.

**Table 5:** Trust in sources for vaccine information

Source^a^	Did NOT intend to be fully vaccinated (n=40), n (%)			Unsure of full vaccination intentions (n=27), n (%)			Intended to be fully vaccinated with no doses received (n=8), n (%)			Fully vaccinated OR intended to be with 1 dose received (n=661), n (%)	
	Not at all/ little	Moderately/a lot	Not at all/ little		Moderately/a lot	Not at all/ little		Moderately/a lot	Not at all/ little		Moderately/a lot
Family	31 (77.5)	8 (20.0)	21 (77.8)		6 (22.2)	2 (25.0)		6 (75.0)	386 (58.4)		269 (40.7)
Friends	32 (80.0)	6 (15.0)	22 (81.5)		4 (14.8)	4 (50.0)		4 (50.0)	445 (67.3)		206 (31.2)
Primary care provider/doctor	33 (82.5)	7 (17.5)	11 (40.7)		16 (59.3)	1 (12.5)		7 (87.5)	51 (7.7)		606 (91.7)
Pharmacist	33 (82.5)	7 (17.5)	14 (51.9)		13 (48.1)	1 (12.5)		7 (87.5)	86 (13.0)		568 (85.9)
Community leaders	35 (87.5)	0 (0)	22 (81.5)		3 (11.1)	3 (37.5)		4 (50.0)	409 (61.9)		194 (29.3)
Local news	34 (85.0)	0 (0)	24 (88.9)		2 (7.4)	4 (50.0)		3 (37.5)	402 (60.8)		204 (30.9)
National news	35 (87.5)	0 (0)	22 (81.5)		3 (11.1)	2 (25.0)		5 (62.5)	310 (46.9)		300 (45.4)
Social media	36 (90.0)	0 (0)	24 (88.9)		2 (7.4)	2 (25.0)		5 (62.5)	539 (81.5)		73 (11.0)
Celebrities	34 (85.0)	0 (0)	24 (88.9)		2 (7.4)	3 (37.5)		4 (50.0)	576 (87.1)		36 (5.4)

^a^There are no responses that are not shown in the table.

The survey also included open-ended responses related to reasons for vaccination intentions, barriers, and facilitators. Most of these responses corroborated trends seen in the quantitative data. In those who did not intend to be vaccinated, other notable reasons for not getting vaccinated included the lack of Food and Drug Administration (FDA) approval and the politics and related political pressures surrounding vaccination. Many people in this cohort also noted that “nothing” would make them choose to get fully vaccinated. In those who intended to be fully vaccinated or were fully vaccinated, a major reason noted for their choice was to prevent the spread of COVID-19 and protecting themselves and others. Lastly, in those who had been fully vaccinated, some noted no concerns with vaccination, while others noted the new vaccine, speed of development, effectiveness, and allergic reactions as concerns.

## Discussion

### Principal Findings

The project survey results showed a significant difference in characteristics and influences between those who were COVID-19 vaccine-hesitant (refusing or delaying) and those who had been vaccinated. Similar to national surveys [24,25], there were differences across gender and political preferences, with a greater percentage of men and Republicans in the did not intend to be fully vaccinated cohort versus the vaccinated cohort. Likewise, COVID-19 risk perceptions among those not planning vaccination are lower [24]. Such characteristics, as well as others identified in Table 1, are especially relevant given the demographic characteristics of Alaska and Idaho. For example, more Alaskans and Idahoans politically identify as Republican than Democrat or Independent [26,27].

Although the sample size for respondents in the unsure or intended to be vaccinated cohorts were smaller, these individuals may be especially important to target in vaccination efforts. Addressing vaccine safety, transparency, and sources of information may encourage some individuals to get vaccinated. More information about how well the vaccine works was also seen as an important facilitator. Conveying this information to the lay public can be difficult given the technical and scientific details related to vaccine mechanism of action and how vaccine effectiveness data are calculated, reported, and interpreted [28].

The safety of vaccines (particularly long-term side effects) was noted as a barrier in 80% (28/35) of respondents in the unsure and intended to be vaccinated cohorts. Although case reports have revealed legitimate safety concerns with vaccination (eg, myocarditis and pericarditis with messenger RNA vaccines), risks of severe adverse events are still low [29]. Furthermore, risks of severe adverse events are even higher during and after SAR-CoV-2 infection [30]. A frequent message from the Centers for Disease Control and Prevention has been that COVID-19 vaccines are safe and effective; however, this messaging is not tailored and does not address individual-specific concerns [31]. At least 75% (27/35, 77%) of survey respondents unsure of or delaying vaccination (but intended to be vaccinated) indicated that having enough information about the vaccine that is specific to their health conditions was also a barrier. Therefore, communication needs to also be personalized. It may also be worthwhile to explore ways that local influencers can share their personal experiences or even health systems sharing local data on demographics or characteristics of those vaccinated and their outcomes.

### Comparison With Prior Work

Although much previous related work has been done on this topic, the results presented here are unique given the focus on 2 rural states with continued lower vaccination rates. The identified barriers can be utilized with other resources to facilitate vaccination. Along with addressing individual-specific concerns related to vaccine safety and side effects, national organizations have also made recommendations on word choices to improve vaccine acceptance [32]. A 2020 survey by the de Beaumont Foundation (n=1400) found that family was an especially important motivator for vaccination [32]. Therefore, when discussing the benefits of vaccination, focusing on family may be more helpful than focusing on the country, community, or friends. Although the de Beaumont Foundation data suggest that some individuals may be motivated to receive vaccination for their family, results from the VACCINE project survey indicated that being told by a trusted source to get the COVID-19 vaccine was not a facilitator in most cohorts. Furthermore, family members were only seen as a trusted source of vaccine information by ~20% (14/67, 21%) of respondents in the did not intend to be vaccinated and unsure of vaccination cohorts. However, 75% (6/8) of respondents in the intending to be vaccinated cohorts stated that family was a trusted source, but these results may be skewed due to the lower sample size.

For those unsure or intended to be vaccinated, health care personnel (primary care provider or doctor and pharmacist) were the most trusted source for vaccine information. Health care providers can leverage their position to provide a strong recommendation for vaccination, which has been shown in the past to increase likelihood of vaccination against influenza [31,33-35]. Professionals play a key role in influencing the decision to receive the influenza vaccine. Information about influenza and its vaccine needs to be combined with improvements in service provision if overall target uptake rates of 70% (65% in those aged 65 years and over) are to be achieved [33,34]. Of concern is vaccine misinformation (and disinformation) especially from health care providers [36,37], which has been discussed in recent news outlets. Misinformation can impact intention to vaccinate [38] and has been identified by the US Surgeon General as an urgent public threat [39].

### Limitations

The landscape of the COVID-19 pandemic is rapidly changing, which may impact some of the findings from this cross-sectional work. Since the VACCINE survey was disseminated in mid-June, the proportion of the circulating virus as the Omicron variant increased from 6.3% to 78.4% (as of March 2022), and new variants have arisen. In August 2021, the FDA approved the first COVID-19 vaccine [40]. Since this time period, there have also been increases in vaccination requirements (eg, as seen in President Biden’s COVID-19 Action Plan). Although these national changes have been significant, vaccination rates in Alaska and Idaho are still dismal and below the national average. There continues to be a need to address barriers contributing to vaccine hesitancy in these rural states. Because this work focused specifically on respondents from these 2 states, the broader generalizability to other populations may be limited. Other limitations of this work include a small sample size, especially for those who intended to be fully vaccinated with no doses received, and the use of Facebook advertisements to recruit participants such as this may introduce response bias.

### Conclusions

Efforts to counter vaccine misinformation, address hesitancy, and increase confidence continue to be underway to increase COVID-19 vaccination rates [41]. It is important that targeted approaches are taken in diverse communities (eg, rural areas). The project survey results from 2 rural states indicate that recognition of individual characteristics may influence vaccine choices. However, these individual characteristics represent only a starting point in delivering tailored messages that should come from trusted sources to address vaccination barriers.

## Appendix

Multimedia Appendix 1 Study survey.

Multimedia Appendix 2 Age distribution.

## Abbreviations

FDA: Food and Drug Administration
SAGE: Strategic Advisory Group on Experts
SARS-CoV-2: severe acute respiratory syndrome coronavirus 2
WHO: World Health Organization
