# Supplementary material for: Influences, Barriers, and Facilitators to COVID-19 Vaccination: Cross-sectional Survey on Vaccine Hesitancy in 2 Rural States
Source: JMIR Form Res. 2022 Dec 1;6(12):e39109. doi: 10.2196/39109 (PMC9718362; doi:10.2196/39109)
Supplement: Multimedia Appendix 1 [file formative_v6i12e39109_app1.docx]

VACCINE: COVID-19 Survey

Start of Block: Block 1. Introduction

Q1.1 Faculty at Idaho State University would like to invite you to participate in a survey research study funded by Merck to better understand the factors influencing your decision to receive or not receive the COVID-19 Vaccine. We want to know about your perspectives and opinions regarding safety and efficacy of the COVID-19 vaccine. We want to understand the key factors, including people, that you believe influence the decision to receive or not receive the COVID-19 vaccine. The opinions and experiences collected from this survey may help us better understand the needs of you and your loved ones so we can better support your healthcare decisions. 
 This project is being conducted by Renee Robinson from Idaho State University (ISU) Doctor of Pharmacy Program in Anchorage Alaska. Participation in this project is voluntary and your participation in this survey will not be disclosed to others. If you agree to participate in this project, you would complete a 10-minute survey to better understand your perspectives and opinions regarding safety and efficacy of the COVID-19 vaccine, and the key factors, including people, that you believe influence the decision to receive or not receive the COVID-19 vaccine. 
 Participating in this survey will not benefit you directly, it will not impact your access to the COVID-19 vaccine.  We do not envision any significant risks related to participation. Survey responses will be collected and kept on a password-protected computer and any printed copies will be kept in a locked file cabinet in Dr. Robinson’s locked office and only project staff will be able to read responses. All information will be destroyed after completion of the study and publication of results. For those that complete the survey and provide their name and contact information, 20 names will be randomly chosen using a random number generator to receive a $100 gift card.
 If you have any questions about the focus group or corresponding project, please contact Dr. Renee Robinson at robiren2@isu.edu or (907) 786-6233. If you have questions about your rights as a research participant, please contact Ralph Baergen, Institutional Review Board Manager, ISU Institutional Review Board (208)-282-2179 or baerralp@isu.edu.

Q1.2 Are you 18 years of age or older?

- Yes
- No

Skip To: End of Survey If Are you 18 years of age or older? = No

Q1.3 What state do you live in?

- Alaska
- Idaho
- Another state

Skip To: End of Survey If What state do you live in? = Another state

Q1.4 **Fully vaccinated means you got one dose of the Johnson & Johnson COVID-19 vaccine OR two doses of the Moderna or Pfizer COVID-19 vaccines.**

Q1.5 Have you been fully vaccinated with the COVID-19 vaccine?

- No, and I do not plan to be
- No, and unsure if I will be
- No, but I plan to be
- No, I got one of two doses BUT I do NOT plan to get my second dose
- No, I got one of two doses AND I plan to get my second dose
- Yes, I got one dose of the Johnson & Johnson vaccine OR two doses of the Moderna or Pfizer vaccines

End of Block: Block 1. Introduction

Start of Block: Block 2. Barriers/Facilitators (Not Vaccinated or Partially/Do Not Plan 2nd)

Display This Question:

If Have you been fully vaccinated with the COVID-19 vaccine? = No, and I do not plan to be

Or Have you been fully vaccinated with the COVID-19 vaccine? = No, and unsure if I will be

Or Have you been fully vaccinated with the COVID-19 vaccine? = No, I got one of two doses BUT I do NOT plan to get my second dose

Q2.1 Why do you choose to not get fully vaccinated?

________________________________________________________________

________________________________________________________________

________________________________________________________________

________________________________________________________________

________________________________________________________________

| Page Break |  |
| --- | --- |

Display This Question:

If Have you been fully vaccinated with the COVID-19 vaccine? = No, and I do not plan to be

Or Have you been fully vaccinated with the COVID-19 vaccine? = No, and unsure if I will be

Or Have you been fully vaccinated with the COVID-19 vaccine? = No, I got one of two doses BUT I do NOT plan to get my second dose

Q2.2
How much do the following prevent you from getting fully vaccinated?

|  | Not at all | A little | A moderate amount | A lot |
| --- | --- | --- | --- | --- |
| Having enough information about the vaccine that I trust |  |  |  |  |
| Having enough information about the vaccine in my language |  |  |  |  |
| Having enough information on short-term vaccine side effects (those that can happen within one-week of getting it) |  |  |  |  |
| Having enough information on long-term vaccine side effects (those that can happen weeks or months after getting it) |  |  |  |  |
| Having enough information about the vaccine that is specific to my health conditions |  |  |  |  |

Display This Question:

If Have you been fully vaccinated with the COVID-19 vaccine? = No, and I do not plan to be

Or Have you been fully vaccinated with the COVID-19 vaccine? = No, and unsure if I will be

Or Have you been fully vaccinated with the COVID-19 vaccine? = No, I got one of two doses BUT I do NOT plan to get my second dose

Q2.3 How much do the following prevent you from getting fully vaccinated?

|  | Not at all | A little | A moderate amount | A lot |
| --- | --- | --- | --- | --- |
| The process of scheduling a vaccine appointment |  |  |  |  |
| The possible side effects from the vaccine |  |  |  |  |
| The time it takes to get the vaccine |  |  |  |  |
| The time off I might need to take from daily responsibilities if I got side effects from the vaccine |  |  |  |  |

Display This Question:

If Have you been fully vaccinated with the COVID-19 vaccine? = No, and I do not plan to be

Or Have you been fully vaccinated with the COVID-19 vaccine? = No, and unsure if I will be

Or Have you been fully vaccinated with the COVID-19 vaccine? = No, I got one of two doses BUT I do NOT plan to get my second dose

Q2.4 How much do the following prevent you from getting fully vaccinated?

|  | Not at all | A little | A moderate amount | A lot | Not applicable |
| --- | --- | --- | --- | --- | --- |
| I need somebody to watch my kids |  |  |  |  |  |

Display This Question:

If Have you been fully vaccinated with the COVID-19 vaccine? = No, and I do not plan to be

Or Have you been fully vaccinated with the COVID-19 vaccine? = No, and unsure if I will be

Or Have you been fully vaccinated with the COVID-19 vaccine? = No, I got one of two doses BUT I do NOT plan to get my second dose

Q2.5 It is possible that those that receive the vaccine will still need "booster" shots. A booster shot is another dose of the vaccine to help your body fight the infection. 


How much does knowing you might need more shots discourage you from getting fully vaccinated?

- Not at all
- A little
- A moderate amount
- A lot

| Page Break |  |
| --- | --- |

Display This Question:

If Have you been fully vaccinated with the COVID-19 vaccine? = No, and I do not plan to be

Or Have you been fully vaccinated with the COVID-19 vaccine? = No, and unsure if I will be

Or Have you been fully vaccinated with the COVID-19 vaccine? = No, I got one of two doses BUT I do NOT plan to get my second dose

Q2.6 What (if anything) would make you choose to get fully vaccinated?

________________________________________________________________

________________________________________________________________

________________________________________________________________

________________________________________________________________

________________________________________________________________

| Page Break |  |
| --- | --- |

Display This Question:

If Have you been fully vaccinated with the COVID-19 vaccine? = No, and I do not plan to be

Or Have you been fully vaccinated with the COVID-19 vaccine? = No, and unsure if I will be

Or Have you been fully vaccinated with the COVID-19 vaccine? = No, I got one of two doses BUT I do NOT plan to get my second dose

Q2.7 How much would the following encourage you to get fully vaccinated?

|  | Not at all | A little | A moderate amount | A lot |
| --- | --- | --- | --- | --- |
| If somebody I trust tells me to do it |  |  |  |  |
| If I interact with other people who are at high risk of getting really sick from COVID-19 |  |  |  |  |
| If people around me get the vaccine |  |  |  |  |

Display This Question:

If Have you been fully vaccinated with the COVID-19 vaccine? = No, and I do not plan to be

Or Have you been fully vaccinated with the COVID-19 vaccine? = No, and unsure if I will be

Or Have you been fully vaccinated with the COVID-19 vaccine? = No, I got one of two doses BUT I do NOT plan to get my second dose

Q2.8 How much would the following encourage you to get fully vaccinated?

|  | Not at all | A little | A moderate amount | A lot |
| --- | --- | --- | --- | --- |
| There is more information about how well the vaccine works |  |  |  |  |
| I can get the vaccine at my primary care provider's office |  |  |  |  |
| I can get the vaccine somewhere close to me |  |  |  |  |

Display This Question:

If Have you been fully vaccinated with the COVID-19 vaccine? = No, and I do not plan to be

Or Have you been fully vaccinated with the COVID-19 vaccine? = No, and unsure if I will be

Or Have you been fully vaccinated with the COVID-19 vaccine? = No, I got one of two doses BUT I do NOT plan to get my second dose

Q2.9 How much would the following encourage you to get fully vaccinated?

|  | Not at all | A little | A moderate amount | A lot |
| --- | --- | --- | --- | --- |
| I am paid to do it |  |  |  |  |
| It is required for work |  |  |  |  |
| It is required for travel |  |  |  |  |
| I no longer have to wear a mask most of the time |  |  |  |  |

End of Block: Block 2. Barriers/Facilitators (Not Vaccinated or Partially/Do Not Plan 2nd)

Start of Block: Block 3. Barriers/Facilitators (Not Vaccinated/Plan to OR Partially/Planned 2nd)

Display This Question:

If Have you been fully vaccinated with the COVID-19 vaccine? = No, but I plan to be

Or Have you been fully vaccinated with the COVID-19 vaccine? = No, I got one of two doses AND I plan to get my second dose

Q3.1 What concerns (if any) do you have with getting fully vaccinated?

________________________________________________________________

________________________________________________________________

________________________________________________________________

________________________________________________________________

________________________________________________________________

| Page Break |  |
| --- | --- |

Display This Question:

If Have you been fully vaccinated with the COVID-19 vaccine? = No, but I plan to be

Or Have you been fully vaccinated with the COVID-19 vaccine? = No, I got one of two doses AND I plan to get my second dose

Q3.2 How much do the following prevent you from getting fully vaccinated?

|  | Not at all | A little | A moderate amount | A lot |
| --- | --- | --- | --- | --- |
| Having enough information about the vaccine that I trust |  |  |  |  |
| Having enough information about the vaccine in my language |  |  |  |  |
| Having enough information on short-term vaccine side effects (those that can happen within one-week of getting it) |  |  |  |  |
| Having enough information on long-term vaccine side effects (those that can happen weeks or months after getting it) |  |  |  |  |
| Having enough information about the vaccine that is specific to my health conditions |  |  |  |  |

Display This Question:

If Have you been fully vaccinated with the COVID-19 vaccine? = No, but I plan to be

Or Have you been fully vaccinated with the COVID-19 vaccine? = No, I got one of two doses AND I plan to get my second dose

Q3.3 How much do the following prevent you from getting fully vaccinated?

|  | Not at all | A little | A moderate amount | A lot |
| --- | --- | --- | --- | --- |
| The process of scheduling a vaccine appointment |  |  |  |  |
| The possible side effects from the vaccine |  |  |  |  |
| The time it takes to get the vaccine |  |  |  |  |
| The time off I might need to take from daily responsibilities if I got side effects from the vaccine |  |  |  |  |

Display This Question:

If Have you been fully vaccinated with the COVID-19 vaccine? = No, but I plan to be

Or Have you been fully vaccinated with the COVID-19 vaccine? = No, I got one of two doses AND I plan to get my second dose

Q3.4 How much do the following prevent you from getting fully vaccinated?

|  | Not at all | A little | A moderate amount | A lot | Not applicable |
| --- | --- | --- | --- | --- | --- |
| I need somebody to watch my kids |  |  |  |  |  |

| Page Break |  |
| --- | --- |

Display This Question:

If Have you been fully vaccinated with the COVID-19 vaccine? = No, but I plan to be

Or Have you been fully vaccinated with the COVID-19 vaccine? = No, I got one of two doses AND I plan to get my second dose

Q3.5
Why do you choose to get fully vaccinated?

________________________________________________________________

________________________________________________________________

________________________________________________________________

________________________________________________________________

________________________________________________________________

| Page Break |  |
| --- | --- |

Display This Question:

If Have you been fully vaccinated with the COVID-19 vaccine? = No, but I plan to be

Or Have you been fully vaccinated with the COVID-19 vaccine? = No, I got one of two doses AND I plan to get my second dose

Q3.6 How much do the following encourage you to get fully vaccinated?

|  | Not at all | A little | A moderate amount | A lot |
| --- | --- | --- | --- | --- |
| If somebody I trust tells me to do it |  |  |  |  |
| If I interact with other people who are at high risk of getting really sick from COVID-19 |  |  |  |  |
| If people around me get the vaccine |  |  |  |  |

Display This Question:

If Have you been fully vaccinated with the COVID-19 vaccine? = No, but I plan to be

Or Have you been fully vaccinated with the COVID-19 vaccine? = No, I got one of two doses AND I plan to get my second dose

Q3.7 How much do the following encourage you to get fully vaccinated?

|  | Not at all | A little | A moderate amount | A lot |
| --- | --- | --- | --- | --- |
| There is more information about how well the vaccine works |  |  |  |  |
| I can get the vaccine at my primary care provider's office |  |  |  |  |
| I can get the vaccine somewhere close to me |  |  |  |  |

Display This Question:

If Have you been fully vaccinated with the COVID-19 vaccine? = No, but I plan to be

Or Have you been fully vaccinated with the COVID-19 vaccine? = No, I got one of two doses AND I plan to get my second dose

Q3.8 How much do the following encourage you to get the COVID-19 vaccine?

|  | Not at all | A little | A moderate amount | A lot |
| --- | --- | --- | --- | --- |
| I get paid to do it |  |  |  |  |
| It is required for work |  |  |  |  |
| It is required for travel |  |  |  |  |
| I no longer have to wear a mask most of the time |  |  |  |  |

End of Block: Block 3. Barriers/Facilitators (Not Vaccinated/Plan to OR Partially/Planned 2nd)

Start of Block: Block 4. Barriers/Facilitators (Fully Vaccinated)

Display This Question:

If Have you been fully vaccinated with the COVID-19 vaccine? = Yes, I got one dose of the Johnson & Johnson vaccine OR two doses of the Moderna or Pfizer vaccines

Q4.1 What concerns (if any) did you have with getting fully vaccinated?

________________________________________________________________

________________________________________________________________

________________________________________________________________

________________________________________________________________

________________________________________________________________

| Page Break |  |
| --- | --- |

Display This Question:

If Have you been fully vaccinated with the COVID-19 vaccine? = Yes, I got one dose of the Johnson & Johnson vaccine OR two doses of the Moderna or Pfizer vaccines

Q4.2 How much were you worried about the following when you got fully vaccinated?

|  | Not at all | A little | A moderate amount | A lot |
| --- | --- | --- | --- | --- |
| Having enough information about the vaccine that I trust |  |  |  |  |
| Having enough information about the vaccine in my language |  |  |  |  |
| Having enough information on short-term vaccine side effects (those that can happen within one-week of getting it) |  |  |  |  |
| Having enough information on long-term vaccine side effects (those that can happen weeks or months after getting it) |  |  |  |  |
| Having enough information about the vaccine that is specific to my health conditions |  |  |  |  |

Display This Question:

If Have you been fully vaccinated with the COVID-19 vaccine? = Yes, I got one dose of the Johnson & Johnson vaccine OR two doses of the Moderna or Pfizer vaccines

Q4.3 How much did the following make it hard for you to get fully vaccinated?

|  | Not at all | A little | A moderate amount | A lot |
| --- | --- | --- | --- | --- |
| The process of scheduling a vaccine appointment |  |  |  |  |
| The concern for side effects from the vaccine |  |  |  |  |
| The actual side effects from the vaccine |  |  |  |  |
| The time it took to get the vaccine |  |  |  |  |
| The concern for time off from daily responsibilities I might need to take if I got side effects from the vaccine |  |  |  |  |
| The actual time off from daily responsibilities because of side effects from the vaccine |  |  |  |  |

Display This Question:

If Have you been fully vaccinated with the COVID-19 vaccine? = Yes, I got one dose of the Johnson & Johnson vaccine OR two doses of the Moderna or Pfizer vaccines

Q4.4 How much did the following make it hard for you to get vaccinated?

|  | Not at all | A little | A moderate amount | A lot | Not applicable |
| --- | --- | --- | --- | --- | --- |
| Somebody to watch my kids |  |  |  |  |  |

| Page Break |  |
| --- | --- |

Display This Question:

If Have you been fully vaccinated with the COVID-19 vaccine? = Yes, I got one dose of the Johnson & Johnson vaccine OR two doses of the Moderna or Pfizer vaccines

Q4.5
Why did you choose to get fully vaccinated?

________________________________________________________________

________________________________________________________________

________________________________________________________________

________________________________________________________________

________________________________________________________________

| Page Break |  |
| --- | --- |

Display This Question:

If Have you been fully vaccinated with the COVID-19 vaccine? = Yes, I got one dose of the Johnson & Johnson vaccine OR two doses of the Moderna or Pfizer vaccines

Q4.6 How much did the following encourage you to get fully vaccinated?

|  | Not at all | A little | A moderate amount | A lot |
| --- | --- | --- | --- | --- |
| Somebody I trust told me to do it |  |  |  |  |
| I interact with other people who are at high risk of getting really sick from COVID-19 |  |  |  |  |
| People around me got the vaccine |  |  |  |  |

Display This Question:

If Have you been fully vaccinated with the COVID-19 vaccine? = Yes, I got one dose of the Johnson & Johnson vaccine OR two doses of the Moderna or Pfizer vaccines

Q4.7 How much did the following encourage you to get fully vaccinated?

|  | Not at all | A little | A moderate amount | A lot |
| --- | --- | --- | --- | --- |
| There was more information about how well the vaccine works |  |  |  |  |
| I could get the vaccine at my primary care provider's office |  |  |  |  |
| I could get the vaccine somewhere close to me |  |  |  |  |

Display This Question:

If Have you been fully vaccinated with the COVID-19 vaccine? = Yes, I got one dose of the Johnson & Johnson vaccine OR two doses of the Moderna or Pfizer vaccines

Q4.8 How much did the following encourage you to get fully vaccinated?

|  | Not at all | A little | A moderate amount | A lot |
| --- | --- | --- | --- | --- |
| I got paid to do it |  |  |  |  |
| It was required for work |  |  |  |  |
| It was required for travel |  |  |  |  |
| I no longer had to wear a mask most of the time |  |  |  |  |

End of Block: Block 4. Barriers/Facilitators (Fully Vaccinated)

Start of Block: Block 5. Influences

Q5.1 In your opinion, what is your risk of getting COVID-19?

- No risk
- Low risk
- Medium risk
- High risk

Q5.2 In your opinion, what is your risk of getting really sick from COVID-19?

- No risk
- Low risk
- Medium risk
- High risk

Q5.3 Have you had COVID-19?

- No
- Unsure
- Yes, and I have / had only minor or no symptoms
- Yes, and I am / was really sick

Q5.4 Has somebody you know had COVID-19? Select ALL that apply.

- No
- Unsure
- Yes, and they have / had only minor or no symptoms
- Yes, and they are / were really sick
- Yes, and they died from COVID-19

Q5.5 How much do you believe that vaccines work to prevent diseases?

- Not at all
- A little
- A moderate amount
- A lot

Q5.6 How much do you **trust** the following sources to give information about the COVID-19 vaccine?

|  | Not at all | A little | A moderate amount | A lot |
| --- | --- | --- | --- | --- |
| Family |  |  |  |  |
| Friends |  |  |  |  |
| Primary care provider / doctor |  |  |  |  |
| Pharmacist |  |  |  |  |
| Community leaders, please describe: |  |  |  |  |
| Local news, please describe: |  |  |  |  |
| National news, please describe: |  |  |  |  |
| Social media, please describe: |  |  |  |  |
| Celebrities, please describe: |  |  |  |  |
| Other, please describe: |  |  |  |  |

End of Block: Block 5. Influences

Start of Block: Block 6. Demographics

Q6.1 How old are you (in years)?

________________________________________________________________

Q6.2 What is your gender?

- Man
- Woman
- Non-binary / third gender
- Other, please describe: ________________________________________________
- Prefer not to say

Q6.3 What is your race? Select ALL that apply.

- White
- Black or African American
- Alaska Native
- American Indian / Native American
- Asian
- Native Hawaiian or Pacific Islander
- Other, please describe: ________________________________________________
- Prefer not to say

Q6.4 What is your ethnicity?

- Not Hispanic or Latino
- Hispanic or Latino
- Other, please describe: ________________________________________________
- Prefer not to say

Q6.5 What is the 5-digit zip code of where you live?

________________________________________________________________

| Page Break |  |
| --- | --- |

Q6.6 What is your religion? Select ALL that apply.

- Christian
- Protestant ((Baptist, Methodist, Non-denominational, Lutheran, Presbyterian, Pentecostal, Episcopalian, Reformed, Church of Christ, etc.)
- Roman Catholic (Catholic)
- Mormon (Church of Jesus Christ of Latter-day Saints / LDS)
- Jewish
- Muslim (Islam)
- Agnostic (not sure if there is a God)
- Atheist (do not believe in God)
- Nothing in particular
- Other, please describe: ________________________________________________
- Prefer not to say

Q6.7 What is your political preference? Select ALL that apply.

- Republican
- Democrat
- Independent
- Other, please describe: ________________________________________________
- Prefer not to say

Q6.8 What is the highest grade of school you have finished or the highest degree received?

- None
- 1st - 6th grade
- 7th - 9th grade
- High school graduate or GED
- Some college, no degree
- Associate's degree
- Bachelor's degree
- Master's degree
- Professional degree
- Doctoral degree
- Other, please describe: ________________________________________________
- Prefer not to say

Q6.9 What is your current employment status? Select ALL that apply.

- Employed by a private for-profit company or business
- Employed by a private not-for-profit company or business
- Employed by local government
- Employed by state government
- Employed by federal government
- Self-employed in an incorporated or not incorporated business, practice, farm, etc.
- Working without pay
- Student
- Retired
- Not working
- Other, please describe: ________________________________________________
- Prefer not to say

Q6.10 Do you have health insurance?

- No
- Unsure
- Yes
- Prefer not to say

Q6.11 In what area do you work? Select ALL that apply.

- Agriculture, forestry, fishing, and hunting
- Mining, quarrying, and oil and gas extraction
- Construction
- Manufacturing
- Wholesale trade
- Retail trade
- Transportation and warehousing
- Utilities
- Information
- Finance and insurance
- Real estate and rental and leasing
- Professional, scientific, and technical services
- Management of companies and enterprises
- Administrative and support and waste management services
- Educational services
- Health care and social assistance
- Arts, entertainment, and recreation
- Accommodation and food services
- Other public services, except public administration
- Public administration
- Military
- Other, please describe: ________________________________________________
- Not applicable, not working
- Prefer not to say

| Page Break |  |
| --- | --- |

Q6.12 Do you typically receive your flu vaccine?

- No
- Unsure
- Yes
- Prefer not to say

Q6.13 Do you have any allergies that you worry / worried about with getting the COVID-19 vaccine?

- No
- Unsure
- Yes, please describe: ________________________________________________
- Prefer not to say

End of Block: Block 6. Demographics
