# Supplementary figures and images for: Influences, Barriers, and Facilitators to COVID-19 Vaccination: Cross-sectional Survey on Vaccine Hesitancy in 2 Rural States
Source: JMIR Form Res. 2022 Dec 1;6(12):e39109. doi: 10.2196/39109 (PMC9718362; doi:10.2196/39109)

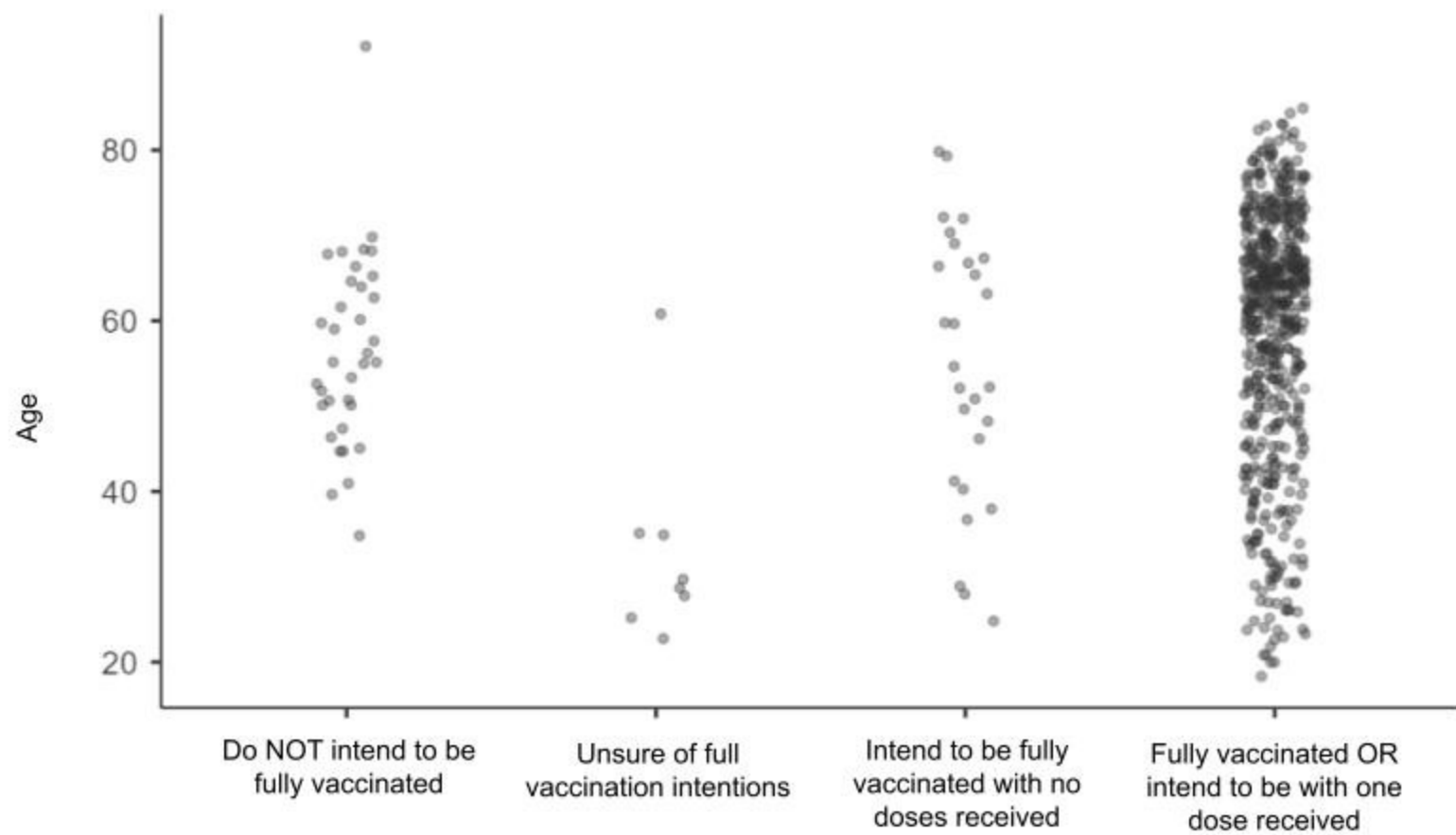

Supplement: Multimedia Appendix 2 [file formative_v6i12e39109_app2.pdf]
